# Supplementary material for: Potential of Higher Resolution Synchrotron Radiation Tomography Using Crystal Analyzer-Based Imaging Techniques for Differential Diagnosis of Human Lung Cancers
Source: Cancers (Basel). 2025 Dec 26;18(1):82. doi: 10.3390/cancers18010082 (PMC12785047; doi:10.3390/cancers18010082)
Supplement: Supplementary file 1 [file cancers-18-00082-s001.zip › Supplementary Figures.pdf]

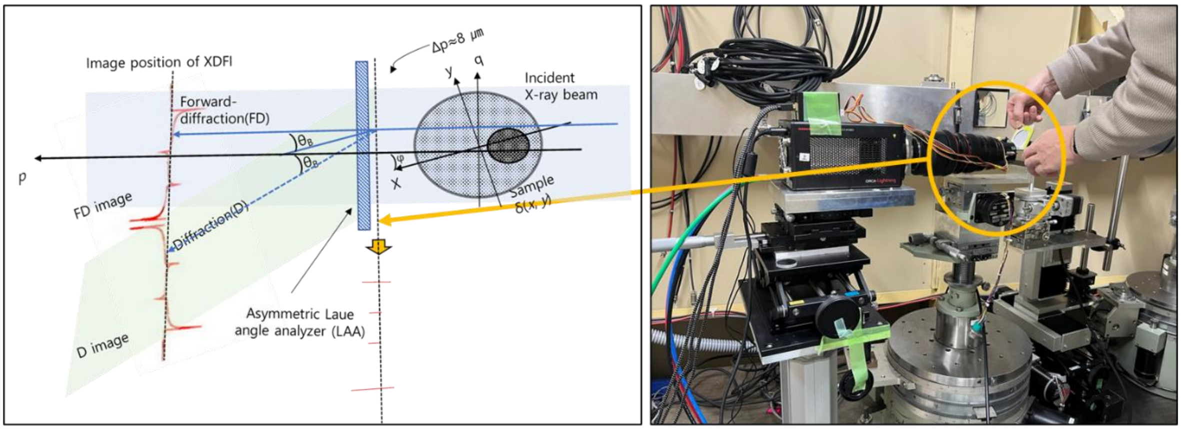

(a) (b)

Figure S1. XDFI imaging system combined with asymmetrically cut single crystal (LAA). (a) Schematic illustration of the asymmetric Laue angle analyzer (LAA) geometry used in crystal-based X-ray dark-field imaging. (b) Positioning of LAA in experimental stage of BL 14B.

The incident synchrotron X-ray beam is first diffracted by the AMC under an asymmetric Bragg condition, generating a broadened and collimated exit beam whose divergence is governed by the asymmetric factor b = sin (θB − α)/sin (θB + α), where θB is the Bragg angle and α is the asymmetry angle of the crystal. As the beam propagates through the specimen, refraction and phase shifts cause angular deviations (φ) proportional to local electron-density gradients. Downstream, the beam is separated by the LAA into forward-diffraction (FD) and analyzer-diffraction (AD) paths, producing complementary refraction-contrast signals. The resulting FD and AD intensity profiles capture sub-microradian angular variations, enabling high-sensitivity phase-contrast imaging of soft-tissue microstructures. This geometric configuration forms the theoretical basis for the refraction-contrast microtomography used in this study. Forward-diffracted rays, captured by the X-ray camera, provided refraction-contrast images representing angular deviations due to specimen structure. The acrylic filter minimized artifacts arising from the shape of sample containers. Specimens were stabilized in cylindrical agarose-filled containers to avoid significant X-ray refraction occurring at the boundary between the specimen and the air, which leads to severe image artefacts.

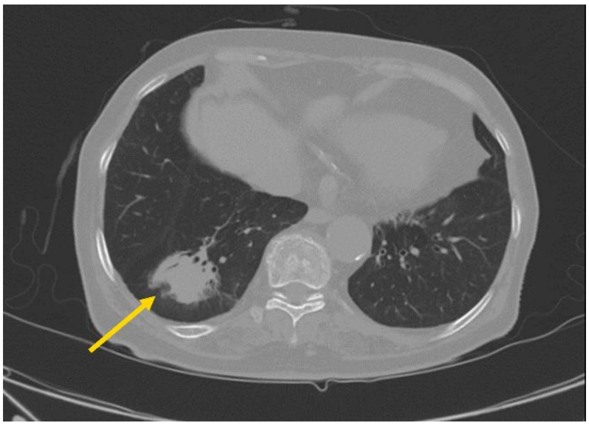

(**a**)

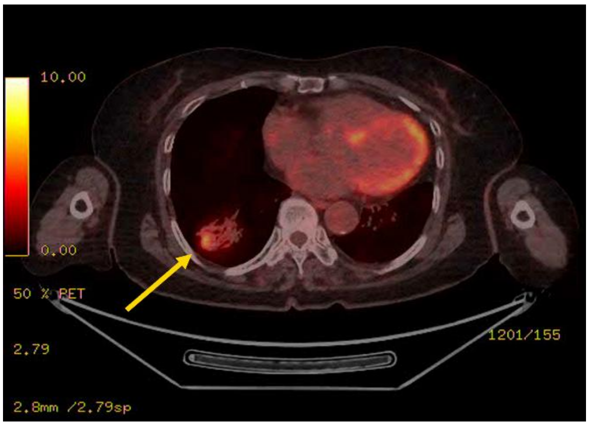

(**b**)

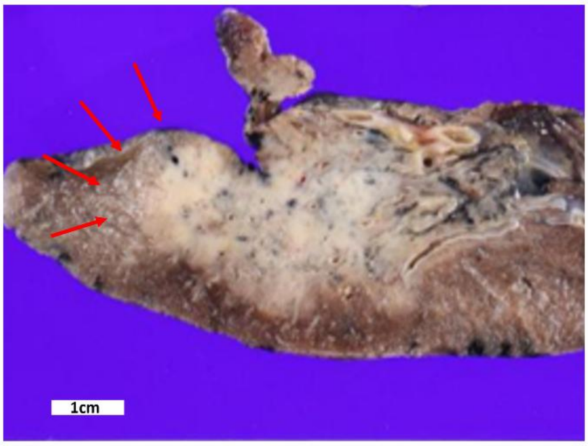

(c)

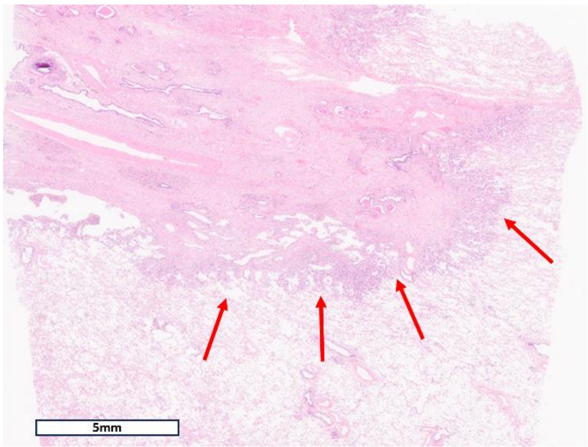

(d)

Figure S2. Clinical and pathological imaging information of specimen #\1, which was resected from primary adenocarcinoma with acinar predominant pattern. (a) A single slice from chest CT (lung window setting). Solid pulmonary mass (3.9 ㎝ in largest dimeter, yellow arrow) with spiculated margin is noted in Rt. Lower lobe. Multiple pores assuming air bronchograms and pleural retraction identified in peripheral area of the main lesion. Ground-glass opacity rim is surrounding the periphery. (b) A transection view from PET-CT in similar level with (a). A hypermetabolic mass (SUVmax value≒6.1, yellow arrow) with pleural tagging is seen. (c) Gross appearance of serially cut resected specimen. The cut surface showed ill-defined yellowish gray solid mass (red arrows). (d) Histologic whole slide scan view under light microscopy (scale bar, 5mm), which findings are corresponding with adenocarcinoma with acinar prominent pattern. Small portion of lepidic component is noted at the transition zone to the normal parenchyma (red arrows.)

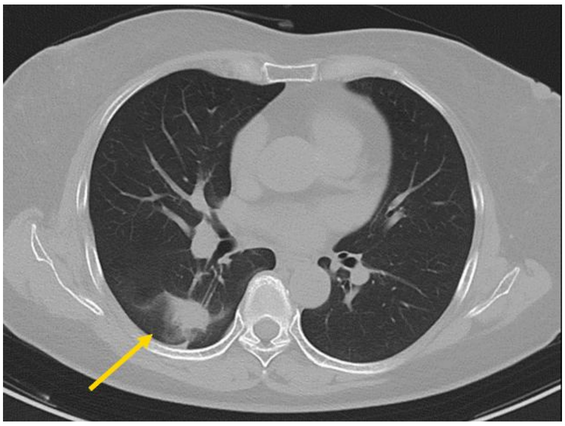
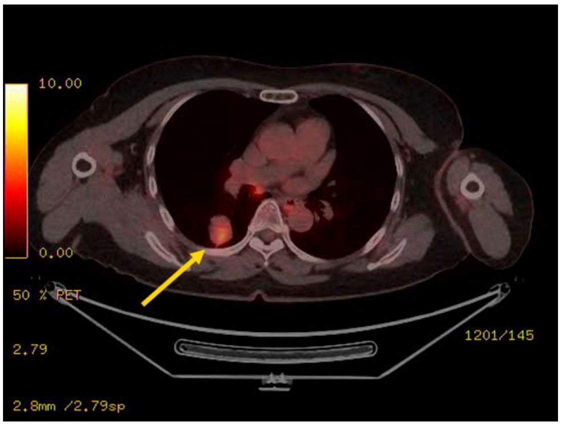

(**a**) (**b**)

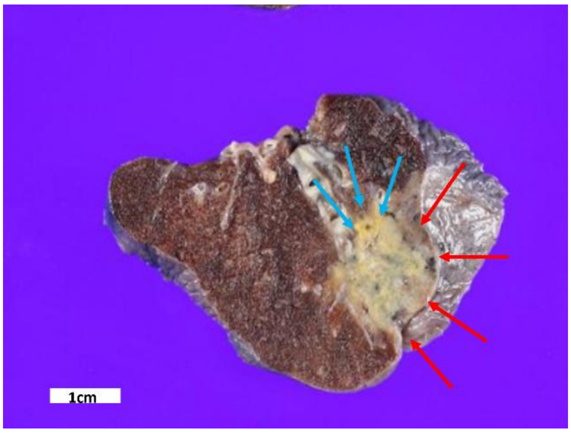

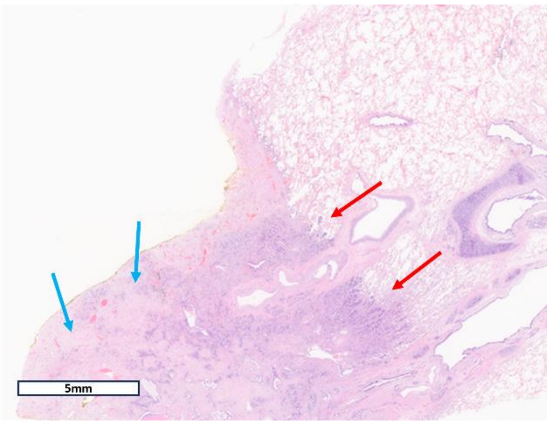

(a) (b)

Figure S3. Clinical and pathological imaging information of specimen 2, which was resected from primary adenocarcinoma with acinar predominant pattern. The specimen was resected after concurrent chemoradiation therapy. (a) A single slice from chest CT (lung window setting). Solid pulmonary mass (2.6 ㎝ in largest dimeter, yellow arrow) with spiculated margin is noted in Rt. Lower lobe. Peripheral ground glass rim was supposed to be pneumonitis due to radiation (yellow arrow). The initial tumor dimeter was 3.2 ㎝ at the similar slice view, but was decreasing after neoadjuvant treatment. (b) A transection view from PET-CT in similar level with (a), and taken before neoadjuvant therapy. A hypermetabolic mass (SUVmax value≒6.5, yellow arrow) with pleural tagging is seen, and a little larger than the tumor (about 3.2 ㎝ in larger diameter) observed in slice (a) (c) Gross appearance of serially cut resected specimen. The cut surface showed ill-defined yellowish gray solid mass (red arrows). Brighter yellowish lesion in medial margin of tumor (blue arrows) is necrosis due to the neoadjuvant treatment effect. (d) Histologic whole slide scan view under light microscopy (scale bar, 5mm) from a lung cancer tissue resected after neoadjuvant CCRT. Necrotic changes expressing as accumulation of pink materials (blue arrows) are corresponding with post-treatment changes. Peripheral lepidic components are also noted at the marginal area between malignancy and normal parenchyma (red arrows).

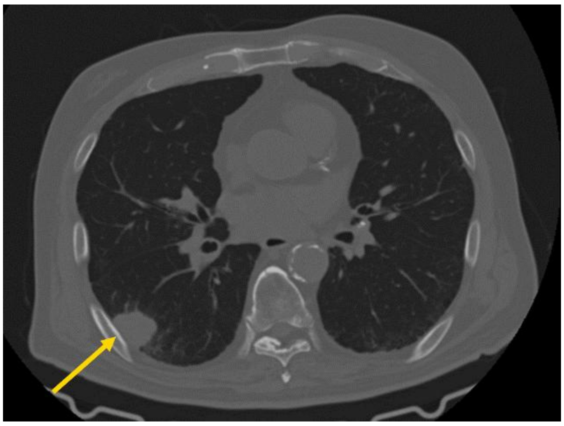

(**a**)

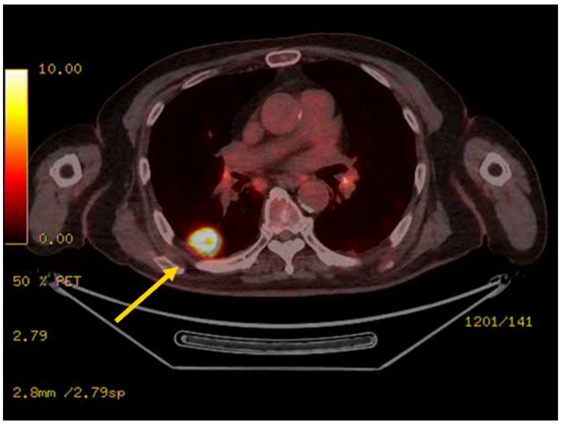

(**b**)

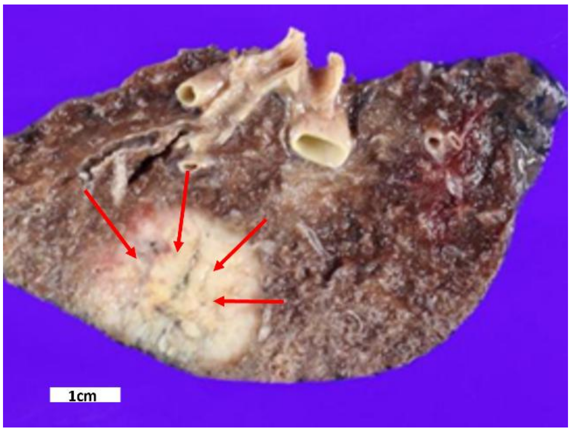

(a)

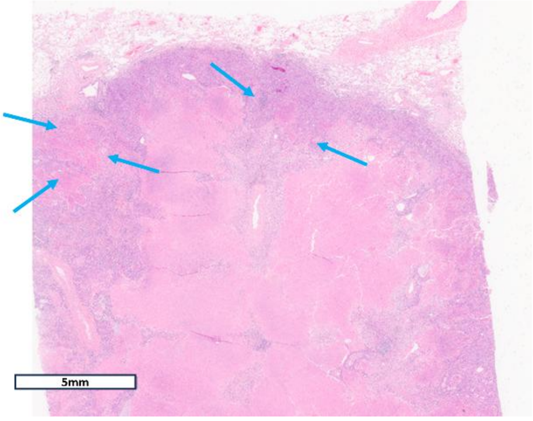

(b)

Figure S4. Clinical and pathological imaging information of specimen 3, which was resected from primary squamous cell carcinoma with keratinizing phenomenon. (a) A single slice from chest CT (lung window setting). Solid pulmonary mass (2.7 ㎝ in largest dimeter, yellow arrow) with relatively round margin, contacting visceral pleura (clinically T2a) is noted in Rt. Lower lobe. A transection view from PET-CT in similar level with (a). A hypermetabolic mass (SUVmax value≒9.5, yellow arrow) with pleural involvement is observed. (c) Gross appearance of serially cut resected specimen. The cut surface showed gritty tan-white nodular lesion with irregular margin contrast to dark brownish normal parenchyma. In the central, pale-yellowish softened lesion is noted, suggesting necrosis or keratinous debris (red arrows). (d) Histologic whole slide scan view under light microscopy (scale bar, 5mm), with H&E staining. Keratinizing pearls are observed inside the broad tumor cell nests (blue arrows). Internal eosinophilic components are correspondence with desmoplastic stromal changes caused by necrosis.

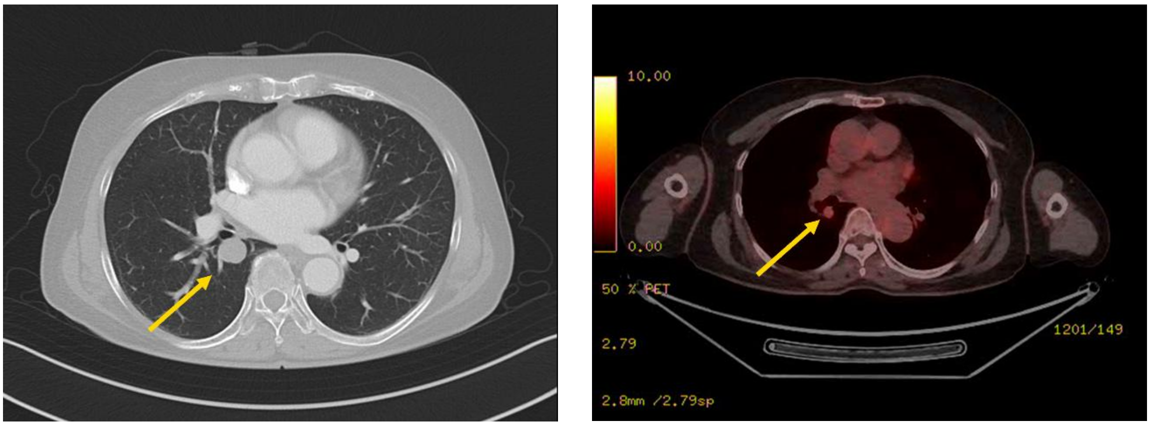

(**a**) (**b**)

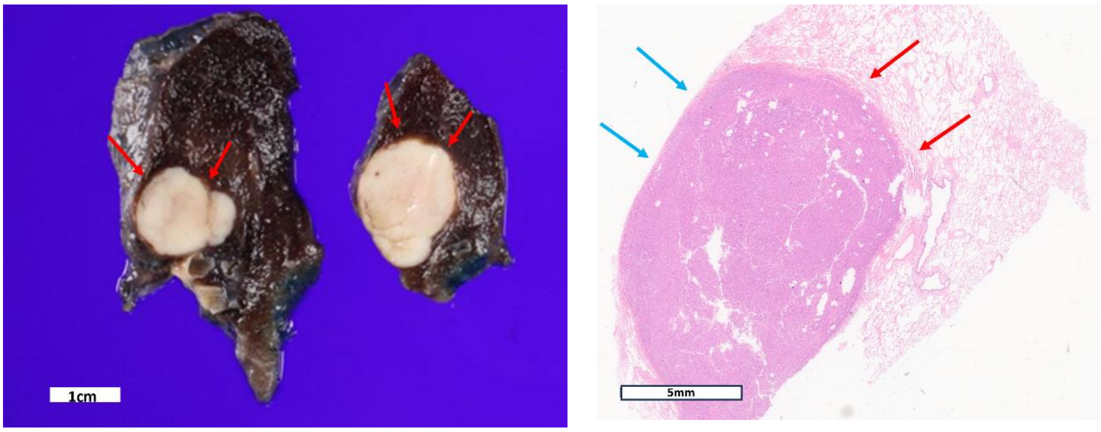

(a) (b)

Figure S5. Clinical and pathological imaging information of specimen 4, which was resected metastatic hepatocellular carcinoma. (a) A single slice from chest CT (lung window setting). Solid homogenous round mass (2.1 ㎝ in largest dimeter, yellow arrow) with round, well discriminated margin is noted in Rt. Lower lobe. A transection view from PET-CT in similar level with (a). A hypermetabolic mass (SUVmx value≒3.5, yellow arrow) is note near the right lower lobe bronchus. (c) Gross appearance of serially cut resected specimen. The cut surface showed well-circumscribed lobulating whitish nodules (red arrows), with smooth and glistening features. (d) Histologic whole slide scan view under light microscopy (scale bar, 5mm), with H&E staining. Homogenous eosinophilic cytoplasm is observed. Sporadic pale pores suggest low cytoplasm and focal necrosis. The tumor lobules protrude toward visceral pleura (blue arrows), but shows no definitive invasion. The tumor neighboring normal parenchyma has thin desmoplastic rim, and compress normal parenchyma (red arrows).
